# Supplementary material for: The basic reproduction number of COVID-19 across Africa
Source: PLoS One. 2022 Feb 25;17(2):e0264455. doi: 10.1371/journal.pone.0264455 (PMC8880647; doi:10.1371/journal.pone.0264455)
Supplement: S1 Appendix — Table A1. List of countries, their population sizes, date of their index case of COVID-19, and the non-pharmaceutical interventions (NPIs) implemented against COVID-19 in the early stage of the disease in the countries with dates. The population sizes are for the year 2020 and were obtained from the worldometer population website [78]. When an interval is specified for the date of NPIs, it means the implementation of the NPIs started on a day within the specified interval. [74–76, 79–100]. Table A2. Same caption as Table A1. [81, 92, 101–121]. (PDF) [file pone.0264455.s001.pdf]

A Appendix

Table A1. List of countries, their population sizes, date of their index case of COVID-19, and the non-pharmaceutical interventions (NPIs) implemented against COVID-19 in the early stage of the disease in the countries with dates. The population sizes are for the year 2020 and were obtained from the worldometer population website [78]. When an interval is specified for the date of NPIs, it means the implementation of the NPIs started on a day within the specified interval.

Country	Population size	Date of First confirmed COVID-19 case	Non-pharmaceutical interventions implemented with date
Nigeria	206,139,589	27-Feb-2020 [79, 80]	**Mar 18 - Apr 10:** Travel restrictions, lockdown of non-essential activities and stay-at-home orders in Abuja, Lagos and Ogun States [74].
Ghana	31,072,940	12-Mar-2020 [81, 82]	**Mar 22:** Travel restrictions [83]. Lockdown was imposed in parts of Accra and Kumasi on Mar 30 [75].
Senegal	16,743,927	01-Mar-2020 [81, 84]	**Mar 14 - 22:** Closure of schools, travel restrictions, and state of emergency [85, 86].
Mali	20,250,833	25-Mar-2020 [87]	**Mar 18:** Travel restrictions, closure of schools, and ban on public gathering [76].
South Africa	59,308,690	05-Mar-2020 [81]	**Mar 27:** National lockdown (see [88, 89] for details).
Zambia	18,383,955	18-Mar-2020 [90]	**Mar 20:** Closure of schools, colleges and universities, and travel restrictions [91].
Namibia	2,540,905	13-Mar-2020 [81, 92]	**Mar 17:** State of emergency, travel restrictions, ban on large gatherings, mandatory quarantine, and lockdown in parts of the country [93].
Malawi	19,129,952	01-Apr-2020 [94]	**Apr 24:** 21-day lockdown [95, 96].
Egypt	102,334,404	14-Feb-2020 [81, 92]	**Mar 15 - 25:** Travel restrictions, closure of schools, workplaces, and lockdown [97].
Tunisia	11,818,619	2-Mar-2020 [81, 92]	**Mar 20:** Travel restrictions, closure of mosque, cafes, and markets [98].
Algeria	43,851,044	25-Feb-2020 [81, 92]	**Mar 9:** International travel restriction, public gathering limitation, quarantine, and lockdown [99].
Morocco	36,910,560	2-Mar-2020 [81, 92]	**Mar 20:** Closure of airports, schools, mosques, businesses (except markets), ban on large gatherings, implement social distancing [100].

Table A2. Same caption as Table A1

Country	Population size	Date of First confirmed COVID-19 case	Non-pharmaceutical interventions implemented with date
Ethiopia	114,963,588	13-Mar-2020 [81,92]	**Mar 16 - 23:** Suspension of schools, sporting events, and public gatherings for 15 days. Closure of night clubs and all land borders [101,102].
Kenya	53,771,296	13-Mar-2020 [81,92]	**Mar 15-25:** Closure of all learning institutions, bars and restaurants, ban on social gathering, travel restrictions, dusk to dawn curfew imposed [103].
Rwanda	12,952,218	14 March 2020 [104]	**Mar 20:** National lockdown and banning of all non-essential travel, labour, and businesses [105].
Sudan	43,849,260	13-Mar-2020 [81,92]	**Mar 16 - 24:** Closure of airports, nationwide curfew and lockdown [106].
Cameroon	26,545,863	6-Mar-2020 [92]	**Mar 18**: Closure of land, air and sea borders, and mandatory mask wearing in public spaces on April 13 [107] .
Chad	16,425,864	19-Mar-2020 [108]	**Apr 13:** Closure of schools and workplaces, and partial lockdown in different regions [109].
Gabon	2,225,734	13-Mar-2020 [81,92]	**Jul 1:** Suspension of visas for all European travelers [110].
Republic of the Congo	5,518,087	10-Mar-2020 [92]	**Mar 31:** Nationwide lockdown [111].
Madagascar	27,691,018	19-Mar-2020 [112]	**Mar 20 - 31:** Suspension of international and regional flights [113]. Lockdown in at least two cities [114].
Comoros	869,601	30-Apr-2020 [115]	**Apr 30:** Curfew imposed, and mandatory wearing of masks outdoors on the whole territory implement on July 8 [116,117]
Mauritius	4,649,658	18-Mar-2020 [118]	**Mar 20:** Nationwide "sanitary" lockdown. Essential services and certain economic services, and minimum public transport are allowed to operate (see [119] for more details).
Cape Verde	555,987	19-Mar-2020 [120]	**Mar 28:** Nationwide state of emergency imposed [121].
